# Clinical Characteristics of Functional Movement Disorders in the Stomatognathic System

**DOI:** 10.3389/fneur.2020.00123

**Published:** 2020-03-13

**Authors:** Kazuya Yoshida

**Affiliations:** Department of Oral and Maxillofacial Surgery, National Hospital Organization, Kyoto Medical Center, Kyoto, Japan

**Keywords:** functional (psychogenic) movement disorders, functional (psychogenic) dystonia, involuntary movement, stomatognathic system, lip, tongue, phenomenology, treatment

## Abstract

**Background:** Functional (psychogenic) movement disorders often have distinguishable clinical features in the orofacial region. Tonic mandibular deviation accompanying ipsilateral downward and lateral lip pulling is the most common phenotype seen in patients with facial functional movement disorders. However, functional movement disorders in the stomatognathic system are underrecognized.

**Objective:** This study aimed to evaluate clinical characteristics and phenomenology in patients with functional movement disorders in the stomatognathic system.

**Methods:** Ten-item inclusion criteria (point range: 0–10) for functional movement disorders in the stomatognathic system was produced, based on previously established criteria for functional movement disorders and general signs of functional facial dystonia, to determine subject inclusion. The criteria included inconsistency, incongruence, and paroxysm in symptoms; rapid onset; distractibility; suggestibility; static course; spreading to multiple sites; spontaneous remission; and lack of sensory tricks. Fifty-eight patients [42 women (72.4%), 16 men (27.6%); mean age: 46.2 years] scored over 7 points on the criteria and were included in further analyses. Characteristic features, including the pattern and site of abnormal movements, were assessed in clinical examination.

**Results:** Frequent items in the scale were inconsistent symptoms (93.1%), incongruous symptoms (91.4%), spreading to multiple sites (89.7%), paroxysmal symptoms (86.2%), and lack of sensory tricks (81%). Sixty percent of patients exhibited a pattern resembling dystonia. Some patients had a combination of organic and functional disease. Common involuntary movements included jaw deviation (74.1%), jaw closing (50%), lip pulling (34.5%), and tongue movement (31%). A functional dystonia phenotype (unilateral lower lip pulling and jaw deviation) was observed in 26 patients (44.8%). Characteristic features of functional stomatognathic movement disorders were rapidly repeating mandibular (lateral or tapping) and tongue movements (27.6%), which fluctuated in speed and direction.

**Conclusion:** In 58 patients with functional movement disorders in the stomatognathic system, the functional dystonia phenotype was observed in 44.8%. Furthermore, 27.6% of patients showed the most characteristic type of functional stomatognathic movement disorders: very fast repeated jaw and/or lingual movements.

## Introduction

Functional (psychogenic) movement disorders are part of a spectrum of functional neurological disorders, which are among the most common causes of neurological disability (1). In recent years, the term “functional” has been used more frequently than “psychogenic” in medical literature (2). The diagnosis of functional movement disorders is challenging. It should rely not on the exclusion of organic diseases or the presence of psychological features, but on the observation of clinical features of the specific movement disorders (3–6). The Diagnostic and Statistical Manual of Mental Disorders does not require the presence of a psychologic stressor as a criterion for diagnosis (7). Tremor, dystonia, myoclonus, and gait disturbance are the most prevalent presentations of functional movement disorders (3–6), which may encompass 5–20% of patients in a movement disorder clinic—functional dystonia being one of the most common (8). Dystonia is characterized by sustained or task-specific muscle contractions that cause abnormal movements and/or postures (9). Diagnosis is based on the characteristic clinical features of focal dystonia, such as task-specificity, stereotypy, sensory tricks, morning benefit, co-contraction, and patients' electromyographic findings (10–13). Oromandibular dystonia is a focal dystonia that affects the masticatory, lower facial, and/or lingual muscles. It can be subdivided into jaw closing dystonia, jaw opening dystonia, lingual dystonia, jaw deviation dystonia, and jaw protrusion dystonia, or a combination of these subtypes (10–14).

Functional facial movement disorders show tonic muscular spasms resembling dystonia, involving the lip, eyelids, perinasal region, and forehead (15–18). The most common phenotype is tonic jaw deviation accompanying ipsilateral downward and lateral lip pulling, seen in 84.3% of patients with facial functional movement disorders involving the craniofacial region (15). Uni- or bilateral orbicularis oculi and platysma contraction are commonly associated (15). Functional movement disorders have rarely been considered from the standpoint of the stomatognathic system, which is an anatomic system comprising the jaws, tongue, lips, teeth, and associated soft tissues. Thus, functional movement disorders in this region are underrecognized. Misdiagnosis of oromandibular dystonia as temporomandibular disorders or a psychogenic disease is frequent. However, the situation in functional movement disorders in the stomatoganathic system must be more serious.

This study aimed to elucidate the phenomenological and clinical characteristics of functional movement disorders in the stomatognathic system.

## Materials and Methods

This is a retrospective study based on the clinical findings of the author in a single institution.

## Patients

One thousand seven hundred and twenty patients with complaints of involuntary movement or contracture of the masticatory, lingual, and/or lower facial muscles visited our department from 2007 to 2019. Structured interviews were carried out for each patient. Clinical features, all symptoms, and medical history were recorded in detail in the medical records. Involuntary movements and the change in symptoms were recorded on video at the first visit and every subsequent visit. Patients who were suspected to have inherited, degenerative, or other neurological diseases were referred to neurologists. Apparent organic diseases such as oromandibular dystonia, temporomandibular disorders, bruxism, dyskinesia, hemifacial spasm, multiple somatization, tremor, tic, myokymia, fibromyalgia, and chronic pain, were excluded from the analysis. Oromandibular dystonia was diagnosed according to the characteristic clinical features of focal dystonia such as stereotypy, task-specificity, sensory tricks, morning benefit, and patients' electromyographic findings (10–14). Temporomandibular disorders, bruxism, and dyskinesia were diagnosed as previously reported (13).

## Inclusion Criteria for Functional Stomatognathic Movement Disorders

To comprehensively select patients with functional movement disorders in the stomatognathic system and minimize the false-positive errors, 10-item inclusion criteria were produced according to previously reported criteria for functional movement disorders (3–6, 19–22) or clinical characteristics in facial functional dystonia (15–18). The criteria included 10 features (rapid onset, static course, paroxysmal symptoms, spreading to multiple sites, spontaneous remission, inconsistent symptoms, distractibility, incongruous symptoms, lack of sensory tricks, and suggestibility) (Table 1). Inconsistency was confirmed when movements varied over time or were changed or suppressed by complex tasks (4). Distractibility was evaluated using a finger-tapping movement at different speeds while counting serial-sevens backward. Incongruence was verified if movements were not present or progressed according to the phenotypic range of established organic movement disorders (4). Suggestibility was examined using a non-physiologic or placebo maneuver. Spontaneous remission was recorded during the follow-up period. If a patient had more than 7 out of 10 characteristics on the criteria, the patient was determined to have functional movement disorders.

**Table 1:** Ten features of inclusion criteria.

	***N***	**%**
Rapid onset	43	74.1
Static course	35	60.3
Paroxysmal symptoms	50	86.2
Spreading to multiple sites	52	89.7
Spontaneous remission	34	58.6
Inconsistent symptoms	54	93.1
Distractibility	39	67.2
Incongruous symptoms	53	91.4
Lack of sensory tricks	47	81.0
Suggestibility	37	63.8

## Diagnosis and Evaluation

After the exclusion of obvious organic disease, 157 patients (110 women and 47 men; mean age ± standard deviation [SD]: 47.2 ± 13.6 years) were evaluated using the rating scale. Fifty-eight patients (42 women and 16 men; mean age: 46.2 ± 13.7 years) were diagnosed with having functional movement disorders after scoring over 7 points on the rating scale (Table 1). The demographic characteristics of the patients are shown in Table 2.

**Table 2:** Demographic characteristics of the patients.

Number of patients [*N*]	58
Age (years) [mean (SD)]	46.2 (13.7)
Sex (women, men) [*N* (%)]	42 (72.4), 16 (27.6)
Duration of symptom (years) [mean (SD)]	4.1 (6.4)
Follow-up duration (months) [mean (SD)]	16.8 (29.1)
**Chief complaints** [***N*** (%)]	
Pain	29 (50.0)
Discomfort	19 (32.8)
Dysarthria	16 (27.6)
Esthetic problem	12 (20.7)
Masticatory disturbance	9 (15.5)
**Patterns of involuntary movements** [***N*** (%)]	
Jaw deviation	43 (74.1)
Jaw closing	29 (50.0)
Lip (pulling or deviation)	20 (34.5)
Tongue (protrusion or deviation)	18 (31.0)
Cervical (platysma or sternocleidmastoid)	17 (29.3)
Jaw opening	13 (22.4)
Around mouth	12(20.7)
Around eye	10 (17.2)
Facial	8 (13.8)
Tremor (lip or palate)	5 (8.6)
Jaw protrusion	2 (3.4)
Others	7 (12.1)
**Characteristic clinical features of organic dystonia** [***N*** (%)]	
Stereotypy	2 (3.4)
Task-specificity	3 (5.2)
Sensory tricks	11 (19.0)
*Intraoral sensory tricks*	11 (19.0)
Chewing gum	8 (13.8)
Foods	3 (5.2)
Candy	1 (1.7)
Tissue paper	1 (1.7)
Gauze	1 (1.7)
*Extraoral sensory tricks*	6 (10.3)
Finger	3 (5.2)
Hand	3 (5.2)
Morning benefit	10 (17.2)
**Other dystonia** [***N*** (%)]	
Cervical dystonia	12 (20.7)
Blepharospasm	9 (15.5)
Writer's cramp	2 (3.4)
Upper limb	2 (3.4)

The pattern and site of abnormal movements, existence of clinical features of organic oromandibular dystonia, and other associated dystonia, were assessed in a clinical examination. The patients were asked for a history of consultations in medical departments and previous diagnoses, the existence of psychiatric diseases, and the duration of medication use. Abnormal jaw movements, including lateral shifts of the mandible, were recorded using Mandibular Kinesiograph (11) (K6 Diagnostic System, Myotronics Research Inc., Seattle, USA).

## Treatments

The patients were treated according to each symptom by means of medication, muscle afferent block therapy (10, 11), botulinum toxin therapy (14, 23, 24), mouth piece (sensory trick splint) (25), myomonitor (transcutaneous electro-neural stimulation) (25), and surgery (26, 27). For mild cases, therapy with anticholinergic or antispasmodic agents, Chinese medicine or other relevant drugs was attempted. For patients with apparent muscle contractions, muscle afferent block therapy (10, 11) by injection of a local anesthetic (0.5% lidocaine, Xylocaine, Aspen Japan, Tokyo, Japan) was attempted on hyperactive muscles. In cases with favorable results, the treatment was continued. However, if the muscle afferent block was effective but the effect was transient, botulinum toxin therapy (Botox®, Allergan, Irvine, USA) was performed (14, 23, 24). If the muscle afferent block therapy showed unfavorable results, then following the patient's consent, they were referred to psychiatrists or acupuncturists. A myomonitor was applied for jaw closing muscle pain (25). Surgical procedures (coronoidotomy) were performed on patients with extremely limited mouth opening (26, 27), under general anesthesia. If these therapies resulted ineffective or gave unsatisfactory responses, sensory trick splints were applied (25). Subjective improvement was evaluated according to a linear self-rating scale ranging from 0 to 100% (10, 11).

This study was performed in accordance with the Declaration of Helsinki under the approval of the institutional review board and ethics committee of Kyoto Medical Center (09-37).

## Results

Table 1 indicates each of the 10 features of the inclusion criteria and their percentages. Frequent items in the scale were inconsistent symptoms (93.1%), incongruous symptoms (91.4%), spreading to multiple sites (89.7%), paroxysmal symptoms (86.2%), and lack of sensory tricks (81%).

## Clinical Features of the Patients

**Figure 1:**
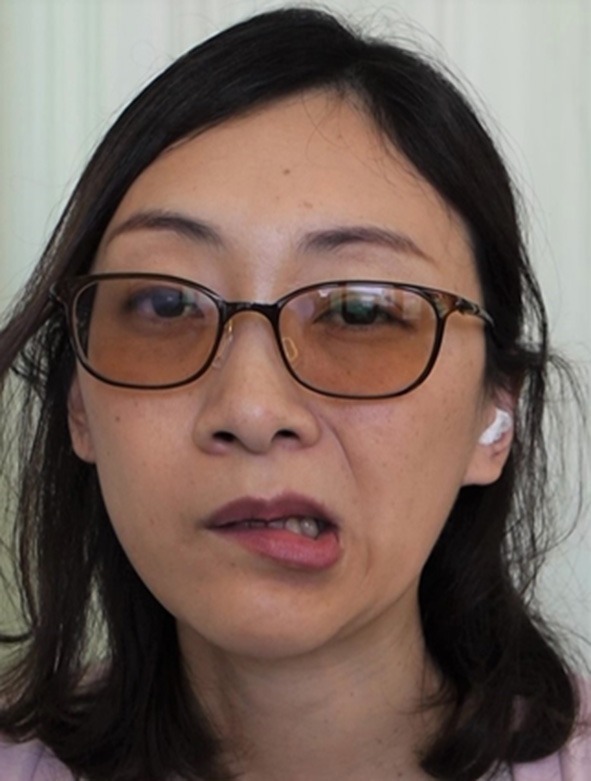
The clinical presentation of functional stomatognathic movement disorders. A case with the well-known “classic” phenotype of orofacial functional dystonia. The patient shows unilateral lower lip pulling, jaw deviation, and platysma contraction (See also Video 1).

**Figure 2:**
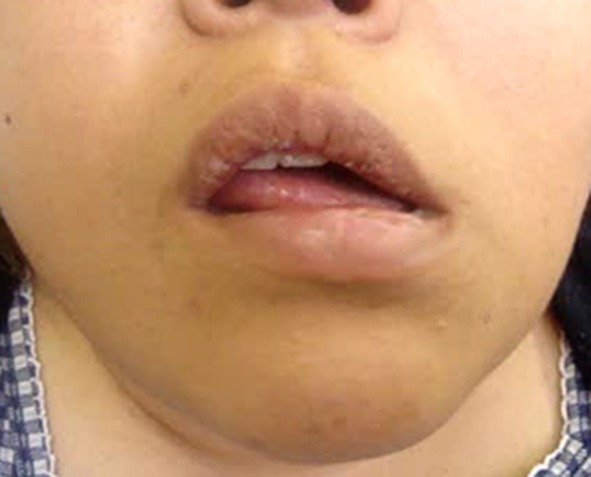
The most characteristic clinical features in functional stomatognathic movement disorders are rapidly repeated mandibular or lingual movements. The direction and speed of the movements occasionally fluctuate from lateral to vertical (See also Video 2).

**Figure 3:**
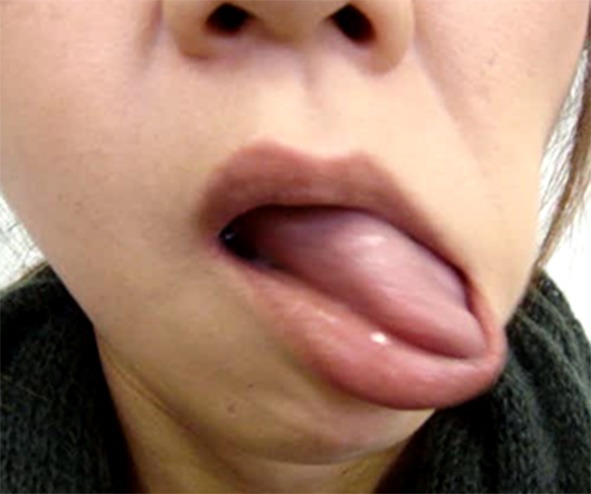
Tongue movement accompanying mandibular movement is complex and bizarre. It does not fall into any of the known movement disorders (See also Video 3).

The results of the analyzed involuntary movements are shown in Table 2. Approximately 60% of patients exhibited dystonia, resembling a pattern of involuntary movement. Other patients showed highly variable, incongruent, and inconsistent movements, which were similar to tremor, tic, and dyskinesia. However, it was impossible to classify them according to established movement disorders. Frequent patterns of involuntary movements included jaw deviation (74.1%), jaw closing (50%), lip pulling (34.5%), and tongue movement (31%) (Table 2). The classic phenotype (unilateral lip pulling and jaw deviation) was observed in 26 (44.8%) patients (22 women and 4 men; mean age 42.0 years) (Figure 1). Characteristic features of functional stomatognathic movement disorders were repeated rapid mandibular (lateral or tapping) and/or tongue movements (Figures 2, 3), which were observed in 13 patients (9 women and 4 men; mean age 48.5 years).

Some patients had a combination of organic and functional diseases. The clinical characteristic features of organic oromandibular dystonia are summarized in Table 2.

## Past History of the Patients

Frequent precipitating events included dental treatments (44.8%) or physical trauma (12.1%) (Table 3). The average lag time between symptom onset and diagnosis of a functional movement disorder was 4.1 ± 6.4 years. The patients had consulted a mean of 6.2 ± 4.6 departments or hospitals (range: 2–23). The visited departments included neurology (75.9%), dentistry (62.1%), oral and maxillofacial surgery (60.3%), psychiatry (55.2%), neurosurgery (36.2%), and acupuncture (29.3%) (Table 3).

**Table 3:** Past history of the patients.

**Precipitating events** [***N*** (%)]	
Dental treatments	26 (44.8)
Tooth extraction	14 (24.1)
Bridge	4 (6.9)
Crown	3 (5.2)
Dental operation	3 (5.2)
Implant treatment	1 (1.7)
Orthodontic treatment	1 (1.7)
Physical trauma	7 (12.1)
Trauma	4 (6.9)
Operation	2 (3.4)
Dislocation	1 (1.7)
Psychological stress	3 (5.2)
Another disease	1 (1.7)
**Visited departments or hospitals** [***N*** (%)]	
Neurology	44 (75.9)
Dentistry	36 (62.1)
Oral and Maxillofacial Surgery	35 (60.3)
Psychiatry	32 (55.2)
Neurosurgery	21 (36.2)
Acupuncture	17 (29.3)
Otorhinolaryngology	12 (20.7)
Orthopedics	5 (8.6)
Pain clinic	3 (5.2)
Internal medicine	3 (5.2)
Rehabilitation	3 (5.2)
Ophthalmology	2 (3.4)
Chiropractic	2 (3.4)
Others	2 (3.4)
**Previous diagnosis** [***N*** (%)]	
Unknown etiology	45 (77.6)
Psychiatric disease	44 (75.9)
Temporomandibular disorders	26 (44.8)
Dyskinesia	21 (36.6)
Bruxism	20 (34.5)
Dystonia	16 (27.6)
Chronic pain	5 (8.6)
Functional dystonia	4 (6.9)
Occlusal problem	3 (5.2)
Trigeminal neuralgia	2 (3.4)
Normal	2 (3.4)
Others	6 (10.3)
**Psychiatric medication** [*N* (%)]	40 (69.0)
**Diagnosis** [***N*** (%)]	
Depression	23 (39.7)
Bipolar disorder	4 (6.9)
Schizophrenia	3 (5.2)
Panic disorder	3 (5.2)
PTSD	3 (5.2)
Others	7 (12.1)
**Duration of medication** (years) [mean (SD)]	14.6 (6.5)
**Prescribed medicine** [***N*** (%)]	
Etizolam	4 (6.9)
Aripiprazole	4 (6.9)
Lorazepam	3 (5.2)
Milnacipran	3 (5.2)
Olanzapine	2 (3.4)
Clotiazepam	2 (3.4)
Bromazepam	2 (3.4)
Lofrazepate	2 (3.4)
Quetiapine	2 (3.4)
Alprazolam	2 (3.4)
Others	6 (10.3)

The patients were diagnosed with or suspected to have conditions of unknown etiology (77.6%), psychogenic disorders (75.9%), temporomandibular disorders (44.8%), bruxism (34.5%), dyskinesia (36.2%), and dystonia (27.6%) (Table 3).

Forty patients (69%) had taken psychiatric medication. In these patients, the mean duration of neuroleptic or tranquilizer use was 14.6 ± 6.5 years (Table 3). Neuroleptic drugs or tranquilizers had been prescribed for depression (39.7%), bipolar disorder (6.9%), and schizophrenia (5.2%) (Table 3). The prescribed drugs included etizolam (6.9%), aripiprazole (6.9%), milnacipran (5.6%), lorazepam (5.2%), and mirtazapine (5.2%) (Table 3).

## Treatments

**Figure 4:**
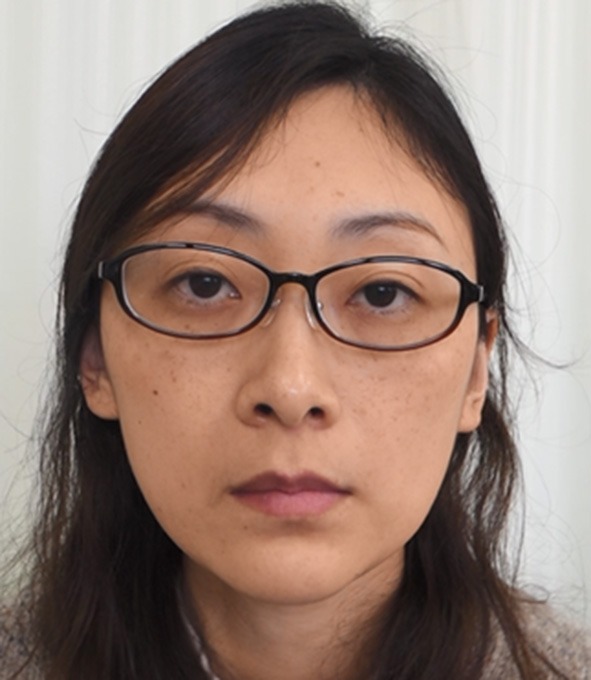
Effects of treatments. After botulinum toxin therapy was applied to the risorius and masseter muscles and platysma for the patient shown in Figure 1, involuntary orofacial movements reduced (See also Video 4).

The results of the treatment methods are summarized in Table 4. The author prescribed baclofen, trihexyphenidyl, clonazepam, tiapride, zolpidem, or Chinese medicine to 34 patients (58.6%). The mean subjective improvement from pharmacotherapy was 15.9%. Botulinum toxin was injected 98 times (range: 1–11 times, mean: 3.5 ± 3.0 times) in 28 patients (48.3%) without significant complications. The injected muscles included the lateral pterygoid, masseter, genioglossus, posterior belly of the digastric, temporalis, risorius, and other muscles, depending on the symptoms of each patient (Table 4, Figure 4). The mean subjective improvement from botulinum toxin injection was 45.2%. Muscle afferent block therapy was performed in 19 patients (32.8%) for a total of 47 times (range: 1–10 times, mean: 2.5 ± 2.7 times), with a mean subjective improvement of 20.6%. Sensory trick splints were made and inserted into the mandibular dental arch of 16 patients (27.6%). The splints were used during daytime and helpful to seven patients (12.1%), with a mean subjective improvement rate of 20.1%. A patient with severe restricted mouth opening underwent coronoidotomy, which resulted in an improvement of the condition. A myomonitor was applied in several patients with jaw elevator muscle pain and brought temporary amelioration of pain. Acupuncture was slightly effective in some patients.

**Table 4:** Results of treatment methods.

**Pharmacotherapy** [*N* (%)]	34 (58.6)
Baclofen	22 (37.9)
Trihexyphenidyl	15 (25.9)
Clonazepam	5 (8.6)
Chinese medicine	5 (8.6)
Tiapride	4 (6.9)
Etizolam	4 (6.9)
Zolpidem	3 (5.2)
Others	3 (5.2)
**Muscle afferent block therapy**	19 (32.8)
Lateral pterygoid (inferior head)	13 (22.4)
Masseter	9 (15.5)
Genioglossus	4 (6.9)
Digastric (posterior belly)	3 (5.2)
Temporalis	2 (3.4)
Risorius	2 (3.4)
Platysma	1 (1.7)
Medial pterygoid	1 (1.7)
Trapezius	1 (1.7)
**Botulinum toxin therapy**	28 (48.3)
Masseter	14 (24.1)
Lateral pterygoid (inferior head)	12 (20.7)
Temporalis	9 (15.5)
Medial pterygoid	5 (8.6)
Genioglossus	4 (6.9)
Zygomatic major	4 (6.9)
Risorius	3 (5.2)
Digastric (posterior belly)	2 (3.4)
Platysma	2 (3.4)
Trapezius	2 (3.4)
Others	4 (6.9)
**Sensory trick splint therapy**	16 (27.6)
**Surgical therapy (coronoidotomy)**	1 (1.7)

## Discussion

This study is the first to evaluate the clinical and phenomenological characteristics of patients with functional movement disorders in the stomatognathic system. This retrospective study was based on the experience of one specialist in involuntary movements in the stomatognathic system at one institution. All patients were diagnosed, evaluated, treated, recorded, and followed-up by the same specialist. Thus, inconsistencies in the diagnosis and evaluation are minimal.

## Diagnosis

Although there are neither specific electrophysiologic tests nor a gold standard for diagnosis, functional dystonia is diagnosed with clinically definite certainty based on available criteria, regardless of any psychiatric symptoms (4, 5). Fahn and Williams (19) introduced four categories of diagnostic certainty: documented, clinically established, probable, and possible. However, the categories of possible and probable functional dystonia are less helpful clinically and only the clinically definite level was suggested to be useful for diagnosis (28). Shill and Gerber (20) suggested revisions to the diagnostic criteria based on a laboratory-supported, clinically definite diagnostic category by means of electrophysiologic findings. Recently, Espay et al. (6) emphasized that a diagnosis will be able to be made in an inclusionary manner by identifying neurological features specific to functional neurological disorders without relying on the presence or absence of psychological stressors or historical clues.

Although authorities on functional movement disorders can differentially diagnose with clinically definite certainty, this is often difficult in the absence of typical clinical features. To comprehensively diagnose patients with functional stomatognathic movement disorders, the author used a rating scale with 10 inclusionary signs as inclusion criteria in this study. Because functional stomatognathic movement disorders can coexist with organic dystonia, dyskinesia, and bruxism in a relatively high number of patients, it was difficult to determine a cut-off point for the scale. The author set a strict inclusion limit (over 7 out of 10 points) to minimize false-positive diagnostic errors, and to evaluate clinical and phenomenological characteristics in clinically definite patients with functional stomatognathic movement disorders. A rating scale is required to confirm reliability and validity on the basis of a standard method for the health measurement scale (13). However, the main purpose of the rating scale described in this study was not to evaluate functional movement disorders, but to comprehensively select patients with this entity from patients with complaints of involuntary movement or contraction. Hence, properties such as internal consistency and test-retest reliability were not analyzed in the present study.

Oromandibular dystonia is often misdiagnosed as temporomandibular disorder, psychogenic disorder, or bruxism (10, 13). Movement disorders in the stomatognathic system constitute a blind spot between medical science and dentistry (13). Although infrequently, neurologists tend to diagnose temporomandibular disorder as oromandibular dystonia, i.e., bruxism as jaw closing dystonia, and internal derangement of temporomandibular joint as jaw deviation dystonia (13). Experience and knowledge of neurology and of dental or oral and maxillofacial surgery are needed to differentiate movement disorders in the stomatognathic system from temporomandibular disorders. Unfortunately, it is practically almost impossible to obtain them simultaneously. The author has treated patients with both involuntary movements and temporomandibular disorders for over 30 years at the departments of both oral and maxillofacial surgery and neurology. Nearly 90% of patients with oromandibular dystonia had either not been diagnosed or were suspected to have oromandibular dystonia (29). Although it is critical to diagnose patients immediately at the primary care level and refer them to appropriate experts, the patients visited 4.1 hospitals for 3.8 years to obtain a diagnosis of oromandibular dystonia (13). In this study, only 5.2% of patients had been suspected to have functional movement disorders. All of them had been diagnosed by experienced neurologists (movement disorder specialists). The patients in the present study visited 6.2 hospitals over 4.1 years. The vast majority of patients with functional stomatognathic movement disorders, who had symptoms solely or mainly in the stomatognathic system, tended to visit at first dentists or oral and maxillofacial surgeons. Frequent diagnoses included unknown etiology, temporomandibular disorders, bruxism, or psychiatric disease (Table 3). The patients were treated without improvement using dental procedures or referred to psychiatrists, leading to iatrogenic harm, unnecessary examinations, and poor outcomes. Patients often abandoned treatment or further consultation. The situation is much more serious in functional oromandibular dystonia than in its organic counterpart. Many patients with functional oromandibular dystonia are neither correctly recognized nor diagnosed. Healthcare workers should pay careful attention to functional stomatognathic movement disorders.

## Phenomenology of Functional Stomatognathic Movement Disorders

The most prevalent chief complaint in this study was pain (50%), while a previous study reported 24.6% of cases complained of painful spasms (15). Dysarthria (27.6%) and masticatory disturbance (15.5%) were also more common in this study than in previous reports (15, 17). This occurred likely because this study evaluated exclusively patients who had symptoms in the stomatognathic region. Depression was observed in 38% of patients in a previous study (15) and in 39.7% of patients in this study.

Frequent precipitating events were dental treatment (44.8%) and physical trauma (12.1%), while physiological stress was only present in 5.2% in this study. Dental treatments or orofacial trauma have been considered to precipitate the onset of oromandibular dystonia in predisposed individuals (30). Peripheral trauma has been associated with the onset of a wide range of movement disorders (31). Minor traumatic events, including dental treatment, might be associated with the occurrence of functional stomatognathic movement disorders.

Combined organic and functional movement disorders can coexist in the same patient. The most common phenotypes of functional facial movement disorders have been recognized as identifiable, such as unilateral lower lip and jaw deviation, or laterocollis with ipsilateral shoulder elevation and contralateral shoulder depression (15, 17, 18). On the other hand, the most characteristic regional phenotype in the stomatognathic system was rapidly repeated mandibular or lingual movements (Figures 2, 3, Videos 2, 3). The abnormal pattern fluctuates in severity, speed, and direction, differing from other organic movement disorders such as dystonia, dyskinesia, tremor, paroxysmal dyskinesia, and tic, not falling into any of the known movement disorders. The inconsistent and incongruent movement pattern agreed very well with functional movement disorders. The pattern was observed in 22.4% of patients.

Most previously reported cases were the classic phenotype of facial functional dystonia accompanying unilateral lip pulling, jaw deviation, and laterocollis (15–18). In this study, 44.8% of patients exhibited this pattern. Fasano et al. (15) reported 84.3% of patients exhibited this phenotype in psychogenic facial movement disorders. In their study, women accounted for 91.8% of patients, with a mean age of 37 years. In the present study, 84.6% of patients were women and the mean age was 42 years in the 26 patients with the classic phenotype. Stone et al. reported that 81% of patients were women (18). In contrast, 72.4% of patients were women in this study. The patients were diagnosed based on the general features of functional dystonia, such as inconsistency and incongruency, using a rating scale as described previously. However, other types of functional movement disorders exist in the stomatognathic region. In a previous study across multiple specialized movement disorder centers, the classic phenotype has been advantageously selected from an enormous number of patients from the database (15). Other types of functional movement disorders in the orolingual region might have been excluded from the analysis. There are probably more patients with functional movement disorders that exhibit not only the well-known classic phenotype but also other types of functional stomatognathic movement disorders.

Sensory tricks are physical movements or positions that can temporarily interrupt dystonia. Patients may be aware of particular sensory tricks that relieve their symptoms. One of the characteristics of functional dystonia is the absence of a sensory trick (3, 6, 15), while it has been reported that 51.4% of patients with organic oromandibular dystonia had sensory tricks (13). In this study, 19% of patients had sensory tricks (Table 2). Morning benefit was observed in 17.2% of patients. Task-specificity and stereotypy were found in a few patients (Table 2). The clinical features of organic dystonia seem to partly overlap those of functional movement disorders.

## Pathophysiology of Functional Stomatognathic Movement Disorders

The etiology of functional movement disorders is multifactorial (22). Gupta and Lang (21) introduced a laboratory-supported, definite diagnostic category according to electrophysiological findings in functional myoclonus and tremor. Electromyography and electroencephalography back-averaging were used for functional myoclonus. Frequency analysis of the electromyogram was applied to document distractibility or entrainment in functional tremor. Their neurophysiologic abnormalities in functional dystonia overlap considerably with those of organic counterparts, with similar abnormalities in somatosensory processing and cortical and spinal inhibition. However, emerging data suggest that there are impairments in regional blood flow and activation patterns on positron emission tomography and functional magnetic resonance imaging (32). Neurobiological abnormalities in functional neurological disorders include hypoactivation of the supplementary motor area and abnormal connectivity with areas that select or inhibit movements that are associated with a sense of agency (6).

We previously attempted to clarify the cortical neurophysiology related to mandibular movements and perception in the stomatognathic system, using electroencephalography (movement-related cortical potentials, contingent negative variation) (33–37), magnetoencephalography (38–40), and near-infrared spectroscopy (39). Because of artifacts arising from masticatory and/or facial activity and involuntary jaw movements, neuroimaging techniques are difficult in patients with involuntary movements in the orofacial region. A few studies have evaluated movement-related cortical potentials, including the Bereitschaftspotential and negative slope in oromandibular dystonia (35, 36). Movement-related cortical potentials amplitude over central and parietal areas for jaw opening and lateral mandibular movements is significantly reduced compared to those in healthy subjects (35). In healthy controls, the movement-related cortical potentials at jaw opening and closing were symmetrically distributed. On the other hand, those of lateral movements showed a predominance in the hemisphere ipsilateral to the direction of the jaw movement (34)—this laterality was not observed in the patient group. These results indicate that there is an abnormal cortical preparatory process for mandibular movements in oromandibular dystonia (35). To date, neurophysiologic studies on functional oromandibular dystonia have not been reported. Further studies are necessary to clarify the pathophysiology, neurophysiology, and etiology of the disorder.

## Treatments for Functional Stomatognathic Movement Disorders

**Figure 5:**
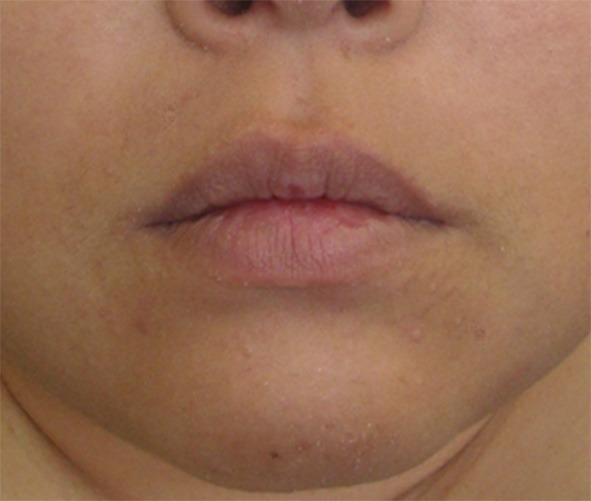
Just after muscle afferent block (injection of 0.5% lidocaine) into the bilateral lateral pterygoid muscle for the patient in Figure 2, rapid lateral jaw movements were completely suppressed (See also Video 5).

Treatment of functional movement disorders should begin with explaining the diagnosis and ensuring the patient's understanding of it (3, 5). Treatments for functional dystonia include antidepressants, psychological therapy in the form of psychodynamic psychotherapy or cognitive behavior therapy, and transcranial magnetic stimulation (3, 6, 32). Generally, therapeutic effects were unsatisfactory in most cases in this study. Psychiatric treatments may have been required in some patients. However, very few psychiatrists or psychotherapists are willing to treat patients with functional movement disorders (41). When patients showed apparent muscle hyperactivity, botulinum toxin therapy helped considerably (Figure 4, Video 4). The author applied muscle afferent block therapy to predict the effect of botulinum toxin therapy (11, 12). For typical cases with jaw or tongue deviation, botulinum toxin should be administered to the lateral pterygoid muscle or tongue. As injection into the muscles can result in severe complications, careful injection based on the local anatomy is required (14, 23, 24). Occlusal splints are effective for patients with a sensory trick in the oral cavity (25). As only 19% of patients had a sensory trick, the splints could relieve symptoms only for a limited amount of patients in this study. Favorable outcomes, including improvement just after muscle afferent block therapy (Figures 2, 5, Videos 2, 5), may be related to suggestibility or the placebo effect in functional movement disorders. The patients have been treated based on the author's experience without providing any level of evidence. Although the setting is clinically difficult, randomized control trials with a larger number of patients will be required to provide evidence of each treatment method. Further development of evidence-based therapeutic strategies is necessary. Furthermore, a multidisciplinary team approach, involving clinicians who can diagnose or treat functional stomatognathic dystonia (neurologist, psychiatrist, neurosurgeon, physiotherapist, psychotherapist, and oral surgeon) is preferable for the diagnosis of and tailored individual therapies for functional oromandibular dystonia.

The author's department specializes in oral and maxillofacial surgery. Therefore, the author solely examined the patients who exhibited symptoms in the stomatognathic system, including the lips, jaws, tongue, and lower face. The author has launched a website for patients with oromandibular dystonia “Involuntary movements of the stomatognathic region” (42), which has been accessed by many visitors from across the world (29). The population in the present report differs considerably from those in previous studies. Patients with hyperkinetic involuntary movements of the stomatognathic system were referred to our department from far away. Many patients who had already abandoned further consultation or treatment might have visited the author's department after informing themselves about involuntary movements of the stomatognathic region via the author's web site (29). At our department, we offer a wide range of multimodal therapies for involuntary movements, including medication, muscle afferent block, botulinum toxin injection, sensory trick splint, and surgery. The selection of subjects was clinic-based in the present study. Therefore, influence of referral bias cannot be excluded. Further studies involving a larger number of population-based samples are required to fully elucidate the clinical and phenomenological aspects of functional stomatognathic movement disorders.

## Conclusion

Functional dystonia phenotype was observed in 44.8% of the 58 patients with functional movement disorders in the stomatognathic system included in this study. Furthermore, 27.6% of patients showed the most characteristic type of functional stomatognathic movement disorder: very fast repeated jaw and/or lingual movements.

## Data Availability Statement

The raw data supporting the conclusions of this article will be made available by the authors, without undue reservation, to any qualified researcher.

## Ethics Statement

The studies involving human participants were reviewed and approved by Institutional Review Board and Ethics Committee of Kyoto Medical Center. The patients/participants provided their written informed consent to participate in this study. Written informed consent was obtained from the individual(s) for the publication of any potentially identifiable images or data included in this article.

## Author Contributions

KY diagnosed and treated all patients, analyzed the results, and wrote the manuscript.

### Conflict of Interest

The author declares that the research was conducted in the absence of any commercial or financial relationships that could be construed as a potential conflict of interest.

## Supplementary Material

Video 1 Note tonic lateral lip pulling, ipsilateral eye closure, and elevation of the contralateral eyebrow.

Video 2 Abnormal lateral jaw movement pattern. The movements fluctuate from lateral to vertical.

Video 3 Note the very fast tongue movement accompanying the mandibular movement. The movement is highly complex, bizarre, and does not fall into any established movement disorder category.

Video 4 Although the lateral cervical movement slightly remains, lip pulling has almost completely disappeared.

Video 5 Rapidly repeated lateral jaw movement abolished just after muscle afferent block therapy of the bilateral lateral pterygoid muscles.
