# Supplementary material for: Clinical Characteristics of Functional Movement Disorders in the Stomatognathic System
Source: Front Neurol. 2020 Mar 13;11:123. doi: 10.3389/fneur.2020.00123 (PMC7082921; doi:10.3389/fneur.2020.00123)
Supplement: Supplementary file 1 [file Table_1.DOCX]

Supplementary Material

**Supplementary Video Legend**

**Video 1.** Note tonic lateral lip pulling, ipsilateral eye closure, and elevation of the contralateral eyebrow.

**Video 2.** Abnormal lateral jaw movement pattern. The movements fluctuate from lateral to vertical.

**Video 3**. Note the very fast tongue movement accompanying the mandibular movement. The movement is highly complex, bizarre, and does not fall into any established movement disorder category.

**Video 4.** Although the lateral cervical movement slightly remains, lip pulling has almost completely disappeared.

**Video 5.** Rapidly repeated lateral jaw movement abolished just after muscle afferent block therapy of the bilateral lateral pterygoid muscles.
